# Associations between adverse childhood experiences and clinical characteristics of eating disorders

**DOI:** 10.1038/srep35761

**Published:** 2016-11-02

**Authors:** S. Guillaume, I. Jaussent, L. Maimoun, A. Ryst, M. Seneque, L. Villain, D. Hamroun, P. Lefebvre, E. Renard, Ph. Courtet

**Affiliations:** 1 Department of Psychiatric Emergency & Acute Care, Lapeyronie Hospital, CHRU Montpellier, Montpellier, France; 2 Inserm, U1061, University of Montpellier, Montpellier, France; 3 Département de Médecine Nucléaire, Hôpital Lapeyronie, CHRU Montpellier, Montpellier, France; 4 Physiologie et Médecine Expérimentale du Cœur et des Muscles, INSERM U1046, UMR9214 CNRS, Université de Montpellier, France; 5 Direction de la Recherche et de l’Innovation, CHRU Montpellier, Montpellier, France; 6 Department of Endocrinology, Diabetes, Nutrition, Montpellier University Hospital, Montpellier, France; 7 INSERM Clinical Investigation Centre 1411, Montpellier, France; 8 Institute of Functional Genomics, CNRS UMR 5203/INSERM U1191, University of Montpellier, Montpellier, France

## Abstract

Patients with eating disorders (EDs) frequently report a history of childhood trauma (CT). We investigated whether certain subtypes of CT are associated with more severe features of EDs, independently of psychiatric comorbidity, and whether they act additively. One hundred and ninety-two patients with DSM-V-defined EDs were consecutively recruited. Five clinical characteristics were assessed: restraint, eating, shape and weight concerns on the EDE-Q, and daily functioning. CT was assessed by the childhood traumatism questionnaire. The clinical features were associated with at least one CT subtype (emotional, sexual or physical abuse, emotional neglect). Multivariate analyses adjusted for lifetime comorbid psychiatric disorders revealed that emotional abuse independently predicted higher eating, shape and weight concerns and lower daily functioning, whereas sexual and physical abuse independently predicted higher eating concern. A dose-effect relationship characterised the number of CT subtypes and the severity of the clinical features, suggesting a consistent and partly independent association between CT and more severe clinical and functional characteristics in EDs. Emotional abuse seems to have the most specific impact on ED symptoms. Last, not all CT subtypes have the same impact but they do act additively.

The lifetime prevalence of eating disorders (EDs) in adults is about 0.6% for anorexia nervosa (AN), 1% for bulimia nervosa (BN), and 3% for binge-eating disorder (BED)^1^. These disorders are often chronic, relapsing, and devastating.

Numerous studies indicate that patients with EDs report a history of childhood trauma more frequently than the general population^2^. A recent meta-analysis, however, found that although childhood abuse is associated with BN and BED, the results remain partly inconclusive for AN^3^. Furthermore, most of these studies have focused on sexual and physical abuse in EDs, with few evaluations of the prevalence of emotional abuse and neglect^3^.

Associations between childhood trauma and EDs have been reported for several subtypes of EDs, although the relationship has been rather heterogeneous among studies^2^. Childhood abuse appears to be frequent in patients with binge episodes or impulsive characteristics^4^,^5^,^6^,^7^,^8^. The findings on the association between childhood abuse and symptoms closely related to body concern, such as body dissatisfaction^9^,^10^ or drive for thinness^11^, have been more discrepant.

The factors underlying the putative association between childhood trauma and EDs remain unclear. Specific genetic and/or epigenetic factors^12^,^13^ and induced emotional dysregulation^14^ may directly promote the emergence of EDs. But the hypothesis of a nonspecific effect cannot be ruled out. Childhood abuse promotes the development of psychiatric disorders, which in turn may lead to eating disorders^7^,^8^,^15^,^16^. These comorbid psychiatric disorders can be mood disorders^17^, post-traumatic stress disorders^18^, substance use disorders^19^ or clinical dimensions related to suicidal behaviour, such as impulsivity or aggressiveness^20^,^21^. It would therefore be useful to determine whether the association between EDs and childhood trauma is independent of the co-occurrence of other psychiatric disorders.

In addition, knowledge is lacking on the types of childhood trauma that are associated with the clinical characteristics and severity of EDs. Some studies suggest greater EDs severity^9^,^22^,^23^ in cases of physical and/or sexual abuse, whereas others do not^4^,^8^,^20^,^24^,^25^. Emotional abuse might also increase EDs severity^6^,^14^,^26^,^27^. Moreover, if child maltreatment is usually associated with a lower quality of life^28^,^29^, to our knowledge, the impact of childhood abuse on quality of life and daily functioning in EDs remains completely unknown. Although it appears that the combination of several types of childhood trauma increases the risk of developing an ED^30^, the data is sparse on how the various types of abuse interact to produce the characteristics of EDs.

In this study, we addressed some of the limitations of prior research by evaluating the independent relationships between the type of childhood trauma and the key features of EDs in a large sample of women diagnosed with AN, BN or BED. We took into account potential confounders of EDs including a lifetime history of psychiatric disorders. We also investigated whether the effects of the trauma subtypes were additive with regard to the clinical characteristics of the EDs. We hypothesised that 1) childhood trauma would be associated with increased severity of the EDs features, 2) this association would be independent of psychiatric comorbidities, and 3) the subtypes of childhood trauma would act additively to increase ED severity.

## Method

### Participants

Overall, 192 female patients with AN (n = 102), BN (n =β64) or BED (n = 26) as defined by the DSM-V were consecutively recruited in an outpatient eating disorders unit of a university hospital in Montpellier, France, between 2013 and 2014. Patients with EDs (or suspected EDs) are sent to this second-line unit (e.g. on caregiver referral) for multidisciplinary assessment, diagnostic confirmation, and organisation of care both for inpatient and outpatient management. The unit serves a population of about 3 million inhabitants. This ancillary study to a larger prospective project was approved by the local ethics committee (CPP Sud Méditerranée IV) and signed informed consent was obtained from all participants (and from parents for underage participants). Research was conducted according to the tenets of the Declaration of Helsinki.

Inclusion criteria were: 15 years or older, French speaking, and diagnosed with one of the three targeted types of EDs. Exclusion criteria were: male (n = 10), absence of an EDs, diagnosed with an eating disorder not otherwise specified (EDNOS) (n = 56), presenting with a physical condition impairing the research (n = 8), and refusal to participate in the study (N = 13).

### Clinical assessment

The multidisciplinary clinical assessment was carried out by experienced psychiatrists, psychologists and nutritionists. The diagnosis was established by consensus using the best-estimated procedure through medical records and information from relatives, the non-standardised clinical assessments of psychiatrists and nutritionists, and standardised measures with the Mini-International Neuropsychiatric Interview (MINI).

In addition, patients completed self-administered questionnaires that assessed the key features of EDs. Five characteristics related to disease severity were assessed using the four subscales of the Eating Disorders Examination Questionnaire (EDE-Q) and the Functioning Assessment Short Test (FAST).

- The EDE-Q^31^ is a 33-item screening tool used to evaluate ED symptoms. It measures disordered eating over a 28-day period and is scored across four subscales that explore the four core clinical dimensions of EDs: eating concern, body shape concern, weight concern, and restraint. The standardised Cronbach’s alpha coefficient was 0.93 for the items of the EDE-Q in this sample.
- The Functioning Assessment Short Test (FAST)^32^ is an interview-administered instrument initially designed for bipolar disorders. It comprises 24 items divided among six areas of functioning: autonomy, occupational functioning, financial issues, interpersonal relationships and leisure time. All items are rated using a 4-point scale and the global score is obtained by summing the scores for each item. The higher the score, the more severe the difficulties are. The standardised Cronbach’s alpha coefficient was 0.91 for the items of the FAST questionnaire in this sample.

### Childhood trauma assessment

Childhood trauma was assessed using the French version of the Childhood Trauma Questionnaire (CTQ)^33^, which retrospectively examines five types of trauma through self-reported assessment: sexual abuse, physical abuse, physical neglect, emotional abuse and emotional neglect. Scores range from 5 to 25 for each type of trauma. In line with the recommendations from Bernstein and Fink, thresholds or cut-off scores were set for each type of trauma at four levels of maltreatment: none, low, moderate and severe. These were further dichotomised as moderate/severe vs. none/low, in order to minimize false identification^34^,^35^ and as previously reported, and were added up to give the total number of childhood traumas^36^.

### Statistical analysis

First, multinomial regression models were used to study the associations among demographic, clinical and trauma features in the AN, BN, BED patients. When a significant relationship was found, two-by-two comparisons were performed to determine whether the diagnostic groups were significantly different (Table 1). A correction for multiple comparisons with the Bonferroni method was used for the two-by-two comparisons.

Second, we examined the relationships between the sociodemographic and clinical variables and the disordered eating measures using several indexes: the EDEQ subscale scores and the FAST global score. As the distribution of these indexes was mostly skewed (Shapiro-Wilk test), they could not be considered as continuous variables. We therefore used the highest tertile as a cutoff (EDE-Q-Dietary Restraint ≥4; EDE-Q-Eating Concern ≥4; EDE-Q-Weight Concern ≥4; EDE-Q-Shape Concern ≥4; FAST total global score ≥28). The associations between the disordered eating indexes and the exposure variables (CTQ dimensions) were quantified with odds ratios (OR) and their 95% confidence interval (CI) (Table 2). Sociodemographic and clinical variables associated with at least one of the disordered eating indexes (with p < 0.10) were included in the logistic regression models to estimate adjusted ORs for exposure variables. When appropriate (i.e. when a CTQ dimension and the diagnostic type were significantly associated with an outcome variable), the interaction terms were tested using Wald χ2 test yielded by the logistic regression model. The significance level was set at p < 0.05. All analyses were performed using SAS statistical software (version 9.4; SAS, Inc., Cary, NC, USA).

## Results

### Sociodemographic and clinical characteristics

In the population of 192 patients, more than half presented with AN and a third presented with BN. In accordance with the three evaluated subtypes, the patients’ current BMI ranged from 10 to 50. The median age was 24.92 years (range: 15.50–65.83). A majority of patients (76.92%) reported university education. The most common lifetime comorbid diagnoses were major depressive disorders followed by anxiety disorders (70.39% and 40.11% respectively). Concerning childhood trauma, 41.67% of patients reported no trauma, while 20.83% reported at least three types of trauma. The most frequent was emotional neglect, reported by 35.94% of patients. Table 1 presents the demographic, clinical and trauma features of the sample according to the ED subtype. Overall, patients suffering from BN experienced more moderate to severe trauma than those suffering from AN. The proportion of patients with physical and sexual trauma did not differ among the three subtypes, whereas emotional abuse and neglect were significantly more prevalent in BN than AN patients (Table 1).

### Relationships between sociodemographic and clinical characteristics according to targeted clinical features

Patients with high restraint (≥4) had more psychiatric disorders like major depressive disorder (p = 0.02), bipolar disorder (p = 0.05) and substance use disorder (p = 0.005); were more likely to present with AN or BN (p = 0.05); and more frequently had a lifetime history of suicide attempts (p = 0.05) than patients with lower restraint. Patients with high eating concern (≥4) had more comorbid substance use disorders (p = 0.05) and were more likely to present with BN or BED (p = 0.003) than patients with lower eating concern. Patients with high weight concern (≥5) were also more likely to present with BN or BED (p = 0.002) and had a more frequent history of suicide attempts (p = 0.04) and comorbid psychiatric disorders (p < 0.01), except for major depressive disorder, than patients with lower weight concern. Patients with high shape concern were more likely to present with BN or BED (p = 0.0001) and to have a lifetime history of suicide attempts (p = 0.005) and all psychiatric comorbid disorders (p ≤ 0.02), except for substance use disorders, than patients with lower shape concern. Patients with poor daily functioning had more anxiety disorders (p = 0.005) but no significant difference was found between the diagnostic subtypes (p = 0.28).

Subsequent analyses were thus adjusted for age, major depressive disorder, bipolar disorder, anxiety disorder, substance use disorder, lifetime history of suicide attempts and diagnostic subtype.

### Crude and adjusted associations between childhood trauma and clinical features of EDs

In the crude associations, childhood emotional abuse increased the severity of all assessed features. Childhood sexual abuse increased the severity of all assessed characteristics except weight concern and set-shifting skills, whereas childhood physical abuse was associated with increased restraint, eating concern and shape concern. Childhood emotional neglect was only associated with higher shape concern (Table 2).

To investigate the impact of psychiatric comorbidities on the relationship between childhood trauma and eating disorders, we performed multivariate analyses adjusted for the associated comorbidities at p < 0.10 with the different disordered eating measures. Emotional childhood abuse was an independent predictor of higher shape and eating concerns and restraint and lower daily functioning, whereas sexual and physical abuses were independent predictors of higher eating concern. Neither physical nor emotional neglect was associated with the clinical features after adjustment for potential confounders.

### Dose-effects of childhood abuse subtypes on clinical characteristics of EDs

**Figure 1:**
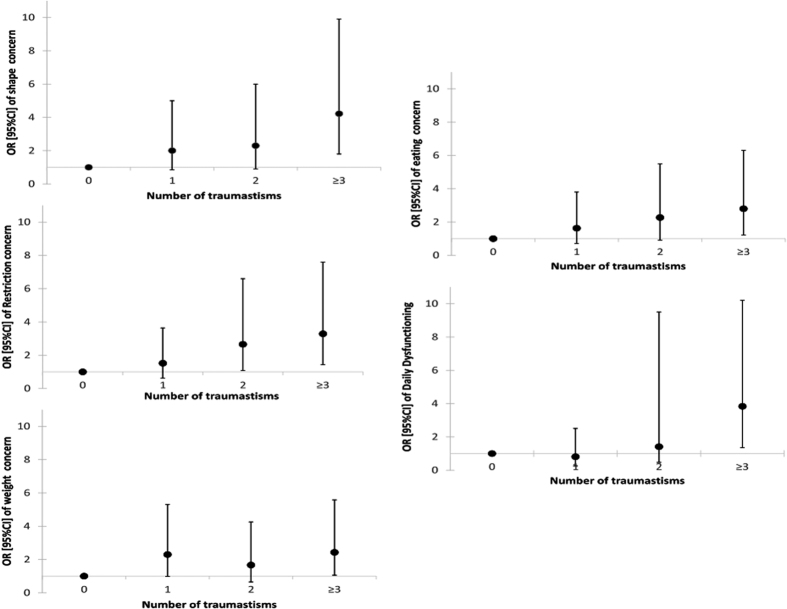
Crude dose-effects of the childhood trauma subtypes on clinical characteristics.

We found a significant crude dose-effect between the types of trauma experienced by the patients and the increased severity for all assessed features (Fig. 1). Nevertheless, after adjustment for age, major depressive disorder, bipolar disorder, anxiety disorder, substance use disorder, lifetime history of suicide attempts and diagnostic type, these dose-effect relationships were only found for daily functioning impairment (p = 0.005), restraint (p = 0.04) and shape concern (borderline significant p = 0.06) (data not shown, available on request) (Fig. 1).

## Discussion

We report an association between the more severe clinical features of EDs and a history of childhood trauma in a large and well-defined sample of patients. We found that childhood abuse was associated with greater severity of the key symptoms of EDs, such as food restriction, weight, shape and food concerns, and daily functioning. These associations seem independent of the co-occurring psychiatric comorbidities particularly concerning emotional abuse. Last, a dose-response effect was observed on most dimensions for the number of traumas in the crude associations.

We built on previous research suggesting that the subtypes of abuse do not have the same impact on EDs. In accordance with some of the studies, our results further suggest that the early life trauma of emotional abuse has the greatest effect on EDs symptoms^26^,^27^. This is in line with prospective studies suggesting that different types of abuse and neglect are equivalently harmful on psychopathological outcomes^37^. Our results also suggest that emotional abuse is most independently associated with EDs, similar to the results found for other psychiatric disorders such as bipolar disorder or addiction^38^,^39^. The nature of the mechanism underlying this association might be the specific impact of this type of abuse on emotional regulation. It could foster the emergence of emotional dysregulation, which in turn could promote the onset and aggravation of ED symptoms. Previous studies on EDs reinforce this hypothesis^6^,^14^,^40^, although longitudinal studies are needed to validate it. Clinically, these results suggest the importance of including emotional abuse in routine clinical assessment, in addition to the more frequently investigated sexual abuse. This is all the more important as our results show an additive effect on the severity of the disorder and than according to worldwide studies, all types of childhood adversities are common (around 36%) with a prevalence of 8% for physical abuse, up to 11,1% for sexual abuse, 8,4% for emotional abuse, and a particularly high prevalence for neglect (up to 18%)^41^,^42^,^43^.

One of our objectives was to study the impact of psychiatric comorbidities on the relationship between early life trauma and eating disorder severity. Numerous studies have shown the role of trauma in the development of psychopathology in adulthood^44^. Rates of psychiatric comorbidities are higher in ED patients with a history of childhood trauma^24^,^45^,^46^. However, few studies have assessed the links between psychiatric comorbidities and the core symptoms of EDs in patients who experienced childhood trauma. Grilo *et al.*^25^ found that maltreatment was not associated with most lifetime psychiatric diagnoses in patients with binge-eating disorders, although specific associations were observed for dysthymic disorder, posttraumatic stress disorder, and alcohol use disorders. Kong and Bernstein^17^ found than depression fully mediated the associations between some forms of childhood trauma and eating psychopathology. Our results suggest that the links between ED severity and physical as well as sexual abuse could be mediated by the presence of one or more psychiatric comorbidities. This is consistent with studies suggesting that sexual and/or physical abuse is more often found in clusters of ED patients with a high rate of psychiatric comorbidities, while clusters with a low proportion of sexual and/or physical abuse present fewer psychiatric comorbidities^5^,^47^. Conversely, our data suggest as previously discussed that the association between ED severity and emotional abuse is independent of co-existing psychiatric disorders.

This study presents several strengths. The sample was consecutively recruited and was large enough to allow us to control the impact of covariables such as psychiatric comorbidities. We used a large set of validated tools to assess trauma and ED characteristics, and several types of abuse and neglect were assessed independently and in combination. To our knowledge, this is the first study to assess the impact of childhood abuse on clinical features such as daily functioning and neuropsychological outcomes, but it nevertheless has some limitations. First, given the relevance of a transdiagnostic approach to EDs^48^,^49^, we voluntarily chose to analyse the three major ED subtypes together although we suspect that examining the potential interactions between the ED subtypes would be a useful future direction. Unfortunately, we could not do so due to sample size constraints. Also, we studied only those patients who were admitted to our second-line unit, which explains the high rates of patients with AN, as well as with previous hospitalizations. Hence, our results can be generalised essentially to patients admitted to a specialised unit. Third, our study is limited by the origin of the information on childhood abuse (retrospective self-reported scale). It should nevertheless be noted that despite its limitations, the CTQ is a widely used and statistically robust questionnaire. Moreover, broad use of the CTQ improves the possibility of comparing results across studies. Last, personality disorders were not assessed, although they are very common in people with histories of childhood abuse and EDs. Some of these disorders via emotion dysregulation might impact the previously suggested link between emotion dysregulation and childhood abuse in EDs^14^,^50^.

Almost all the studies evaluating the relationship between eating disorders and early abuse have been cross-sectional. Future studies should therefore use a longitudinal design, because only this type of study can elucidate the temporal relationships between early life abuse and intermediate factors like the emergence of personality traits or psychiatric comorbidities predisposing to eating disorders. Similarly, we need to define how the types of early life abuse affect the course of an EDs (e.g. risk of switching from one type to another, prognosis) and the responses to the currently available treatments. Also, given the strong associations between suicidal behaviour and both childhood abuse and eating disorders, a better understanding of the interplay of early life abuse, an EDs diagnosis and an increased risk of suicidal behaviour seems crucial. Finally, it should be remembered that all psychiatric disorders (including EDs) are multi-factorial. Thus, early life abuse is a vulnerability factor among others (genetic, epigenetic, cognitive, developmental…). Beside early negative life events, there are also protective factors and events that strengthen the resilience of an individual. There is much need to explore how negative and protective factors interact with each other through the development of an ED. Although difficult to put in practice, this kind of studies are crucial to understand why some subjects with an early abuse will later develop a disorder and others will not.

In conclusion, our results contribute to research on the effects of early life trauma on eating disorders by demonstrating that childhood abuse increases the severity of EDs symptoms. These exacerbations are partially independent of comorbid psychiatric disorders. The subtypes of abuse do not all have the same impact, but they act additively to exacerbate the severity of a wide range of EDs features, including clinical and neuropsychological dimensions and daily functioning.

## Additional Information

**How to cite this article:** Guillaume, S. *et al.* Associations between adverse childhood experiences and clinical characteristics of eating disorders. *Sci. Rep.* **6**, 35761; doi: 10.1038/srep35761 (2016).

**Publisher’s note:** Springer Nature remains neutral with regard to jurisdictional claims in published maps and institutional affiliations.

## Figures and Tables

**Table 1:** Demographic, clinical and trauma features of patients according to eating disorder type: anorexia nervosa (AN), bulimia nervosa (BN), or binge-eating disorder (BED).

	Anorexia nervosaN = 102		Bulimia nervosaN = 64		Binge-eating disorderN = 26		Global p	Post-hoc analysis
	Median [Min-Max]		Median [Min-Max]		Median [Min-Max]			
Age of patient, *in years*	22.63 [15.50–65.83]		26.38 [17.08–63.08]		30.75 [18.58–58.58]		0.02	AN < BED
Duration of the disease, *in years*	2.83 [0.08–40.00]		5.75 [0.42–45.83]		5.79 [0.33–20.75]		0.05	
Current BMI, *in kg/m*^*2*^	16.03 [10.04–20.14]		21.72 [17.35–28.52]		35.05 [16.38–50.99]		<0.0001	AN < BN < BED
Lowest lifetime BMI	14.80 [9.55–17.71]		18.07 [12.02–25.28]		22.53 [10.91–45.04]		<0.0001	AN < BN < BED
Age at the first consultation, *in years*	18.00 [10.00–65.00]		19.00 [10.00–63.00]		22.00 [13.00–52.00]		0.25	
Functioning Assessment Short Test								
Total score	18.00 [2.00–61.00]		21.50 [4.00–54.00]		24.00 [9.00–48.00]		0.50	
Eating Disorders Examination Questionnaire								
Restraint	2.80 [0.00–6.00]		3.60 [0.00–6.00]		1.00 [0.00–4.60]		0.0004	AN, BN < BED
Eating concern	2.60 [0.00–6.00]		4.00 [0.40–6.00]		3.80 [1.20–6.00]		0.0002	AN < BN, BED
Weight concern	3.20 [0.00–6.00]		4.80 [1.20–6.00]		4.80 [3.20–6.00]		<0.0001	AN < BN, BED
Shape concern	3.75 [0.00–6.00]		5.38 [0.63–6.00]		5.25 [3.75–6.00]		<0.0001	AN < BN, BED
	n	%	n	%	n	%		
Educational attainment level (≥12 years)	63	70.00	49	84.48	18	85.71	0.08	
Previous hospitalisation for EDs, Yes	49	49.00	19	31.67	5	19.23	0.009	BED < AN
Lifetime history of psychiatric disorder								
Major depressive disorder	61	64.89	48	80.00	17	68.00	0.14	
Bipolar disorder	10	10.87	14	23.73	6	24.00	0.08	
Anxiety disorder	44	46.81	36	60.00	12	48.00	0.26	
Suicide attempt	14	13.73	20	32.79	4	15.38	0.01	AN < BN
Alcohol/substance dependence or abuse	4	4.30	9	15.25	0	0.00	NA	
Childhood Trauma Questionnaire								
Emotional abuse, moderate/severe	27	26.47	32	50.00	8	30.77	0.009	AN < BN
Physical abuse, moderate/severe	8	7.84	13	20.31	1	3.85	0.03	—
Sexual abuse, moderate/severe	17	16.67	19	29.69	5	19.23	0.14	
Emotional neglect, moderate/severe	28	27.45	32	50.00	9	34.62	0.01	AN < BN
Physical neglect, moderate/severe	25	24.51	20	31.25	8	30.77	0.59	
Number of moderate/severe abuses							0.02	AN < BN
0	54	52.94	15	23.44	11	42.31		
1	18	17.65	17	26.56	6	23.08		
2	14	13.73	12	18.75	5	19.23		
≥3	16	15.69	20	31.25	4	15.38		

**Table 2:** Childhood trauma and clinical features: Model 0: Crude association; Model 1: Adjustment for age, major depressive disorder, bipolar disorder, anxiety disorder, substance use disorder, lifetime history of suicide attempts and diagnostic type.

		Restraint ≥4 (n = 61) vs < 4 (n = 117) OR [95%CI]	Eating concern ≥ 4 (n = 65) vs < 4 (n = 112) OR [95%CI]	Weight concern ≥ 5 (n = 61) vs < 5 (n = 114) OR [95%CI]	Shape concern ≥ 5.38 (n = 59) vs < 5.38 (n = 117) OR [95%CI]	Daily functioning ≥ 28 (n = 40) vs < 28 (n = 79) OR [95%CI]
Sexual abuse	Model 0	2.89 [1.39; 5.98]**	3.28 [1.58; 6.84]**	1.49 [0.71; 3.10]	2.64 [1.28; 5.48]**	2.73 [1.13; 6.60]**
	Model 1	2.05 [0.84; 5.03]	2.67 [1.13; 6.31]*	1.04 [0.42; 2.57]	1.79 [0.71; 4.47]	3.19 [1.08; 9.43]
Physical abuse	Model 0	3.34 [1.28; 8.68]*	2.94 [1.13; 7.64]*	2.04 [0.80; 5.21]	3.48 [1.33; 9.06]*	1.57 [0.50; 4.87]
	Model 1	2.03 [0.64; 5.03]	2.59 [0.85; 7.87]	1.25 [0.39; 3.99]	2.45 [0.76; 7.90]	1.82 [0.45; 7.40]
Emotional abuse	Model 0	2.68 [1.40; 5.13]**	2.49 [1.31; 4.73]**	2.32[1.22; 4.44]*	3.60 [1.85; 6.97]***	3.89 [1.74; 8.66]***
	Model 1	2.78 [1.21; 6.39]*	2.38 [1.10; 5.16]*	1.57 [0.71; 3.47]	2.71 [1.20; 6.12]**	5.82 [2.07; 16.4]**
Emotional neglect	Model 0	1.24 [0.66; 2.34]	1.74 [0.93; 3.25]	1.70 [0.90; 3.21]	2.33 [1.22; 4.43]**	1.34 [0.62; 2.89]
	Model 1	1.39 [0.58; 3.25]	1.43 [0.64; 3.21]	1.17 [0.50; 2.75]	1.74 [0.73; 4.17]	2.04 [0.75; 5.56]
Physical neglect	Model 0	1.79 [0.91; 3.52]	0.96 [0.48; 1.89]	1.12 [0.56; 2.23]	1.49 [0.75; 2.94]	1.66 [0.74; 3.73]
	Model 1	1.98 [0.85; 4.57]	0.75 [0.33; 1.67]	0.94 [0.41; 2.17]	1.27 [0.54; 3.00]	1.70 [0.67; 4.32]

^*^p value < 0.05, ^**^p value < 0.01, ^**^p value < 0.001.
